# Bioinformatics-based analysis reveals elevated MFSD12 as a key promoter of cell proliferation and a potential therapeutic target in melanoma

**DOI:** 10.1038/s41388-018-0531-6

**Published:** 2018-11-01

**Authors:** Chuan-Yuan Wei, Meng-Xuan Zhu, Nan-Hang Lu, Rui Peng, Xuan Yang, Peng-Fei Zhang, Lu Wang, Jian-Ying Gu

**Affiliations:** 1 Department of Plastic Surgery, Zhongshan Hospital, Fudan University, Shanghai, 200032 People’s Republic of China; 2 Liver Cancer Institute, Zhongshan Hospital, Fudan University; Key Laboratory of Carcinogenesis and Cancer Invasion (Fudan University), Ministry of Education, Shanghai, 200032 People’s Republic of China

**Keywords:** Melanoma, Tumour biomarkers

## Abstract

Although recent therapeutic advances based on our understanding of molecular phenomena have prolonged the survival of melanoma patients, the prognosis of melanoma remains dismal and further understanding of the underlying mechanism of melanoma progression is needed. In this study, differential expression analyses revealed that many genes, including AKT1 and CDK2, play important roles in melanoma. Functional analyses of differentially expressed genes (DEGs), obtained from the GEO (Gene Expression Omnibus) database, indicated that high proliferative and metastatic abilities are the main characteristics of melanoma and that the PI3K and MAPK pathways play essential roles in melanoma progression. Among these DEGs, major facilitator superfamily domain-containing 12 (MFSD12) was found to have significantly and specifically upregulated expression in melanoma, and elevated MFSD12 level promoted cell proliferation by promoting cell cycle progression. Mechanistically, MFSD12 upregulation was found to activate PI3K signaling, and a PI3K inhibitor reversed the increase in cell proliferation endowed by MFSD12 upregulation. Clinically, high MFSD12 expression was positively associated with shorter overall survival (OS) and disease-free survival (DFS) in melanoma patients, and MFSD12 was an independent prognostic factor for OS and DFS in melanoma patients. Therapeutically, in vivo assays further confirmed that MFSD12 interference inhibited tumor growth and lung metastasis in melanoma. In conclusion, elevated MFSD12 expression promotes melanoma cell proliferation, and MFSD12 is a valuable prognostic biomarker and promising therapeutic target in melanoma.

## Introduction

Melanoma, the most lethal type of cutaneous malignancy, has increased in incidence and mortality over the past several decades due to its aggressive clinical behavior and propensity for metastasis [1]. Currently, different molecular mechanisms and various biomarkers related to the progression of melanoma have been identified; for example, more than 50% of patients present with a point mutation in the BRAF isoform, which constitutively activates MAPK signaling and plays an important role in melanoma growth, cell proliferation and migration [2]. Similarly, the PI3K pathway, which also plays an essential role in melanoma progression, has been reported to be commonly activated in melanoma cells [3]. Despite the great number of previous studies that have attempted to reveal the molecular mechanism of melanoma progression, the underlying mechanism has not been fully elucidated. Hence, a full understanding of the molecular mechanism promoting melanoma progression is still needed.

Gene expression profiling and bioinformatics analysis are acknowledged to be useful tools for yielding mechanistic insights into cancer development and revealing markers for predicting the prognosis of patients [4, 5]. With the substantial amount of data deposited in public databases, reanalyzing and integrating these available data may provide novel clues for pathological mechanisms of cancer. For examples, Xu et al. [6] identified key genes that could provide potential targets for ovarian cancer diagnosis and treatment, and Bi et al. [7] also found important key genes in bladder cancer by using gene microarray and bioinformatics methods. In melanoma, Robertson et al. [8] identified four molecular and clinical subsets associated with different prognoses using a comprehensive multiplatform analysis.

In this study, we reanalyzed several gene expression profiles from the GEO database. The DEGs were compared between melanoma and normal tissues, and overlapping DEGs were obtained using the Venn online tool. By PPI (protein–protein interaction) network construction [9] and functional enrichment analyses [10], we identified key genes and pathways that may play pivotal roles in tumorigenesis and progression of melanoma. Then, the expression levels and prognostic value of those DEGs were detected by GEPIA (Gene Expression Profiling Interactive Analysis) and were verified by qRT-PCR in our melanoma tissues. By leveraging the analysis results for those DEGs, MFSD12 was selected for the subsequent research. The expression level and role of MFSD12 in melanoma were evaluated, and the correlation between MFSD12 and the major pathological parameters of melanoma was investigated. In addition, the prognostic value of MFSD12 in melanoma was analyzed.

## Results

### Identification of overlapping DEGs in the GSE3189 and GSE31879 profiles

**Fig. 1:**
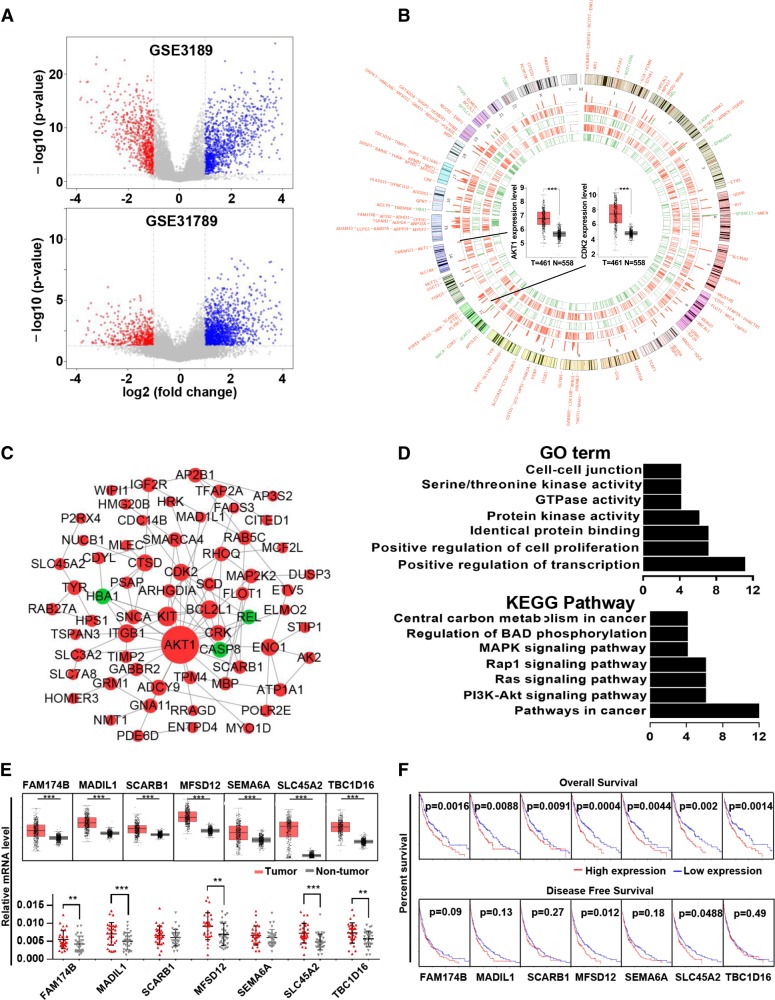
Identifications and analysis of overlapped DEGs in GSE3189 and GSE31879. **a** Volcano plot of the differentially expressed genes in GSE3189 and GSE31879. **b** Tracks 1–4 (inner to outer) show downregulated (green) and upregulated (red) genes in GSE3189 and GSE31879. The outer tracks show all 140 overlapping DEGs, and the inner tracks show two representative genes verified by GEPIA. T tumor, N nontumor. **c** Protein–protein interaction network of the overlapping DEGs. **d** Gene Ontology and KEGG pathway enrichment analyses of the overlapping DEGs. **e** Relative expression of seven DEGs from the GEPIA analysis (upper panel). mRNA expression in our melanoma tissues compared with peritumor tissue (lower panel). **f** Correlation of overall survival with seven DEGs (upper panel). Correlation of disease-free survival with seven DEGs (lower panel). **p < 0.01, ***p < 0.001

There were 45 melanoma and 18 normal samples in GSE3189 and 9 melanoma and 4 normal samples in GSE31879. GSE3189 contained 2390 DEGs, namely, 975 upregulated genes and 1415 downregulated genes; GSE31879 contained 1505 DEGs, namely, 1260 upregulated genes and 245 downregulated genes (Fig. 1a). Of these DEGs, 140 overlapped in GSE3189 and GSE31879 (Fig. 1b). Among these overlapping DGEs, AKT1 and CDK2, which are well recognized as oncogenes, were shown in the center of the Circos plot. Based on the information in the STRING protein query from the public databases, we constructed the PPI network (Fig. 1c) of the overlapping DEGs and found that AKT1 was located in the center of the network; this observation also confirmed the validity of the data. For a more in-depth understanding of the selected DEGs, GO function and KEGG pathway enrichment analyses (Fig. 1d) were performed. GO analysis showed that the DEGs were mainly enriched in the following functions: positive regulation of cell proliferation, protein kinase activity, and GTPase activity; KEGG pathway analysis indicated that the DEGs were particularly enriched in the following pathways: pathways in cancer, the PI3K–AKT signaling pathway, the Ras signaling pathway, and the Rap1 signaling pathway. The above results indicated that most of the overlapping DEGs were related to cancer.

### MFSD12 is upregulated and associated with OS and DFS in GEPIA analysis

For further verification, we submitted the 140 overlapping DEGs to GEPIA to detect the difference in gene expression levels between the tumor and normal samples and the association of these DEGs with prognosis. Expression of FAM174B, MAD1L1, SCARB1, MFSD12, SEMA6A, SLC45A2, and TBC1D16 was found to be upregulated and associated with worse OS in melanoma patients, while only MFSD12 and SLC45A2 were associated with worse DFS for melanoma patients (Fig. 1e, f). Furthermore, we detected the mRNA expression level of these hub genes in our frozen tissues and found that FAM174B, MAD1L1, MFSD12, SLC45A2, and TBC1D16 were significantly upregulated in freshly frozen tumor tissues. By these analyses, we showed that upregulated MFSD12 and SLC45A2, which were associated with OS and DFS, may play an important role in melanoma.

### The expression of MFSD12 is significantly upregulated in melanoma

Interestingly, both MFSD12 and SLC45A2 belong to the major facilitator superfamily, which plays important roles in moving compounds across biological membranes and is associated with a variety of cancers [11]. For example, Park et al. [12] found that SLC45A2 is a promising immunotherapeutic target for melanoma for its high tumor selectivity and reduced potential for autoimmune toxicity. However, little researches have focused on the expression level and role of MFSD12 in cancers, including melanoma. Therefore, we selected MFSD12 for the following study.

**Fig. 2:**
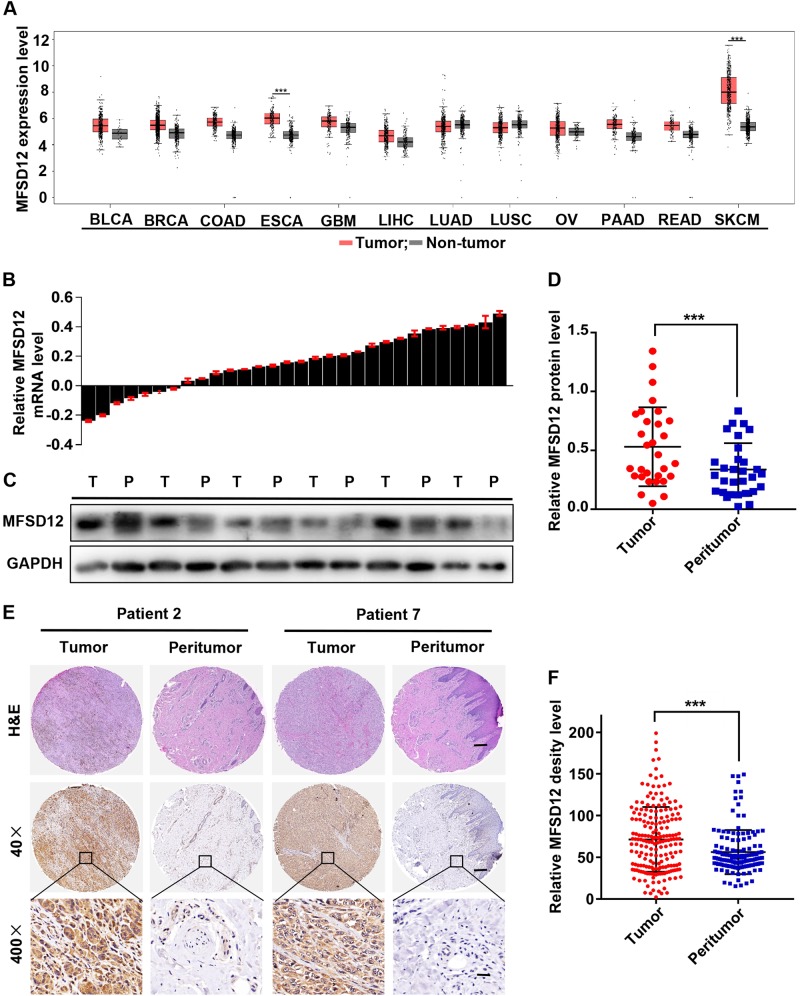
The expression of MFSD12 is significantly upregulated in melanoma. **a** MFSD12 mRNA expression in different cancers. **b** MFSD12 mRNA expression in 30 pairs of melanoma tissues, shown as Log (T/P). T tumor, P peritumor. **c**, **d** MFSD12 protein expression in 30 pairs of melanoma tissues; representative bands are shown. T tumor, P peritumor. **e** Representative images of the TMA stained with H&E and IHC for MFSD12. Scale bar 40×, 500 μm; 400×, 50 μm. **f** MFSD12 protein expression in melanoma and peritumoral tissues was analyzed by densitometry. ^***^p < 0.001

The level of MFSD12 mRNA in different cancers was analyzed with GEPIA (Fig. 2a). The expression of MFSD12 was significantly upregulated in melanoma tissues compared with normal tissues, and the upregulation in melanoma is very conspicuous compared with that in other kinds of cancer. Coincidentally, the MFSD12 mRNA was found to be increased in 23/30 melanoma tissues compared with peritumoral normal tissues (*p* = 0.002, Fig. 2b). Western blot analysis indicated that the expression level of the MFSD12 protein was remarkably higher in melanoma tissues than that in adjacent tumor tissues and six pairs of randomly selected tissues are shown in Fig. 2c, d (*p* < 0.001). Furthermore, we investigated the expression of MFSD12 in a TMA containing 197 melanoma tissue samples. As displayed in Fig. 2e, the representative images revealed that the MFSD12 protein was mainly distributed in the cytoplasm. Through quantification analysis using Image-Pro Plus Chen Y .0, the expression of MFSD12 was found to be noticeably upregulated in melanoma tissues compared with the benign nevus sections (*p* < 0.001, Fig. 2f). Our results indicate that the levels of MFSD12 mRNA and protein are consistently increased in human melanoma tissues.

### MFSD12 promotes the proliferation of melanoma cells in vitro

**Fig. 3:**
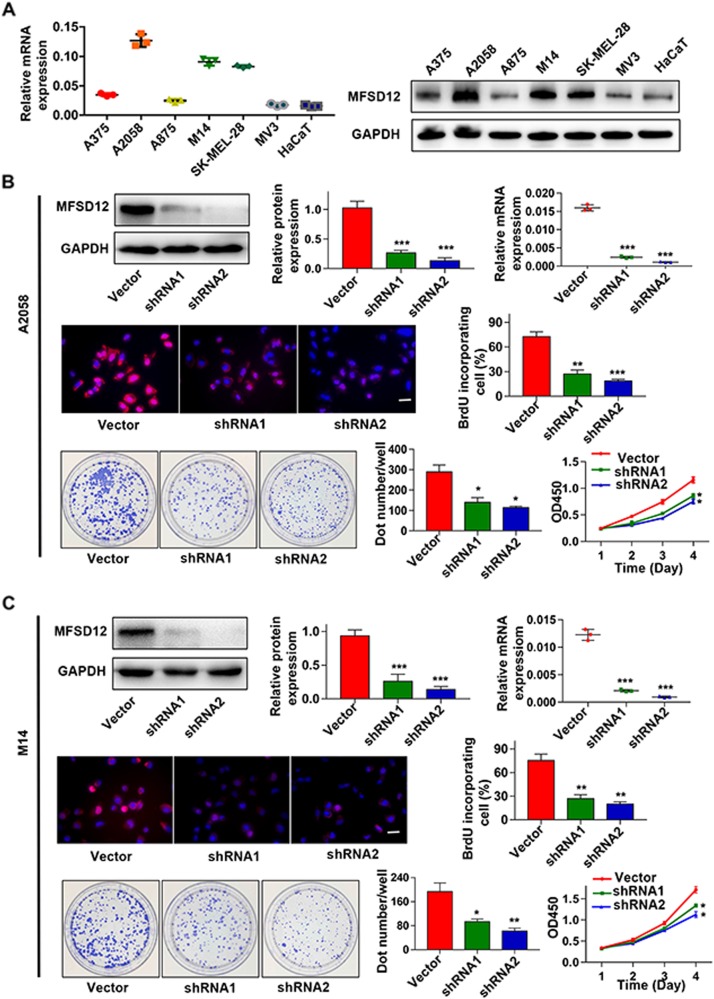
MFSD12 promotes the proliferation of melanoma cells in vitro. **a** Expression analysis of MFSD12 in melanoma (A375, A2058, A875, M14, SK-MEL-28, and MV3) and normal skin (HaCaT) cell lines was performed by western blot analysis and qRT-PCR. **b**–**f** Interference with MFSD12 expression in A2058 cells inhibited cell proliferation as determined by BrdU (middle panel), CCK-8 and colony formation assays (lower panel). **g**–**k** Interference with MFSD12 in M14 cells inhibited cell proliferation as assessed by BrdU (middle panel), CCK-8 and colony formation assays (lower panel). Scale bar, 50 μm. ^*^p < 0.05; ^**^p < 0.01; ^***^p < 0.001

Here, we determined the role of MFSD12 in melanoma. First, the expression of MFSD12 was detected in six melanoma cell lines (A375, A2058, A875, SK-MEL-28, MV3, and M14) and a normal skin cell line (HaCaT) by western blot and qRT-PCR (Fig. 3a). MFSD12 mRNA and protein were found to be obviously upregulated in the melanoma cells especially in A2058 and M14 cells compared with the HaCaT cells. Thus, we knocked down MFSD12 in A2058 and M14 cells by shRNAs. After the downregulation was verified by qRT-PCR and western blot (Fig. 3b, c, g, h), we found that the MFSD12-shRNAs significantly decreased the cell proliferation assessed by BrdU assay (Fig. 3d, i); these results were further confirmed by CCK-8 and colony formation assays (Fig. 3e, f, j, k). MFSD12 upregulation did not influence melanoma cell metastasis (Supplementary Figures S1A and S1B). All of these data indicate that MFSD12 upregulation promotes the proliferation of melanoma cells.

### MFSD12 is involved in the G1-to-S transition of the cell cycle

**Fig. 4:**
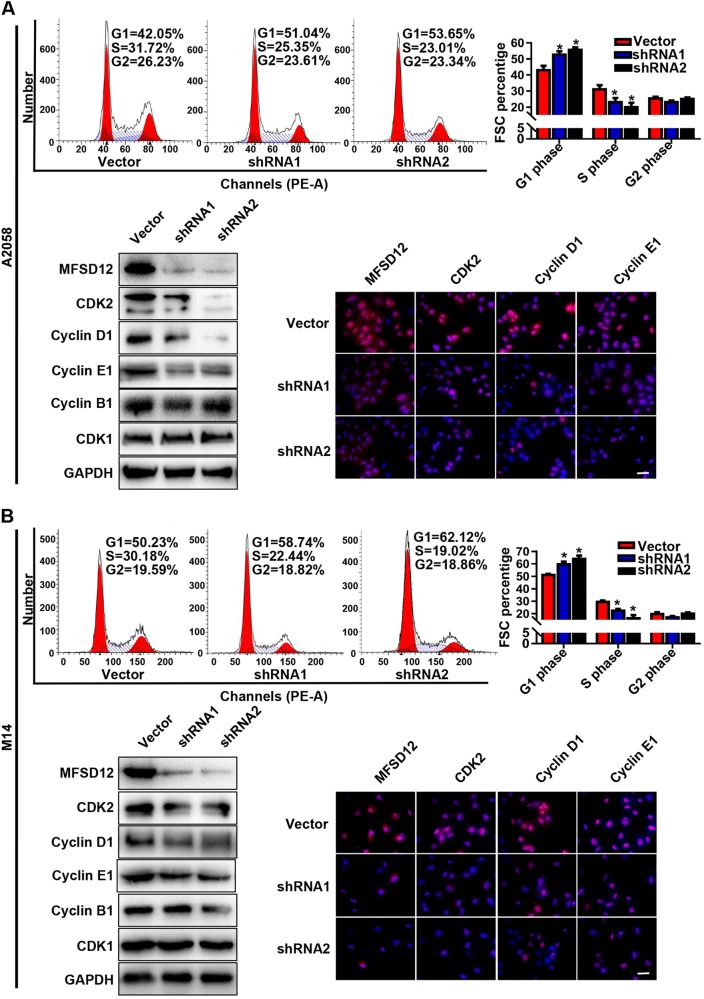
MFSD12 is involved in the G1-to-S phase transition of the cell cycle. **a** Flow cytometric analysis of the indicated cells. **b** Western blot analysis of MFSD12, CDK2, cyclin D1, cyclin E1, cyclin B1, and CDK1 in the indicated cells. **c** Immunofluorescence staining for MFSD12, CDK2, cyclin D1, and cyclin E1 in the indicated cells. **d** Flow cytometric analysis of the indicated cells. **e** Western blot analysis of MFSD12, CDK2, cyclin D1, cyclin E1, cyclin B1, and CDK1 in the indicated cells. **f** Immunofluorescence staining of MFSD12, CDK2, cyclin D1, and cyclin E1 in the indicated cells. Scale bar, 50 μm. *p < 0.05

To investigate the mechanism of the proliferation-promoting function of MFSD12, correlations between MFSD12 and the expression of proliferation biomarkers were analyzed with GEPIA, and positive correlations were detected between MFSD12 and CDK2 (Supplementary Figure S2A, *r* = 0.49, *p* < 0.001) and between MFSD12 and cyclin D1 (Supplementary Figure S2B, *r* = 0.26, *p* < 0.001); both of these genes are biomarkers of the G1-to-S phase transition in the cell cycle. Flow cytometric analysis was then performed and showed that silencing the expression of MFSD12 increased the percentage of cells in G1 phase and decreased the percentage of cells in S phase (Fig. 4a, d), which indicated that MFSD12 might promote the G1-to-S phase transition in melanoma cells. Moreover, western blot analysis and immunofluorescence staining revealed that the cell cycle promoters CDK2, cyclin D1, and cyclin E1 were downregulated in MFSD12-shRNA cells. However, the modification of MFSD12 had no effect on the expression of CDK1 and cyclin B1 (Fig. 4b, c, e, f), both of which are G2-M phase markers. These results indicate that MFSD12 upregulation is involved in the G1-to-S phase transition of the cell cycle.

### MFSD12 regulates melanoma cell proliferation via the PI3K–AKT signaling pathway

**Fig. 5:**
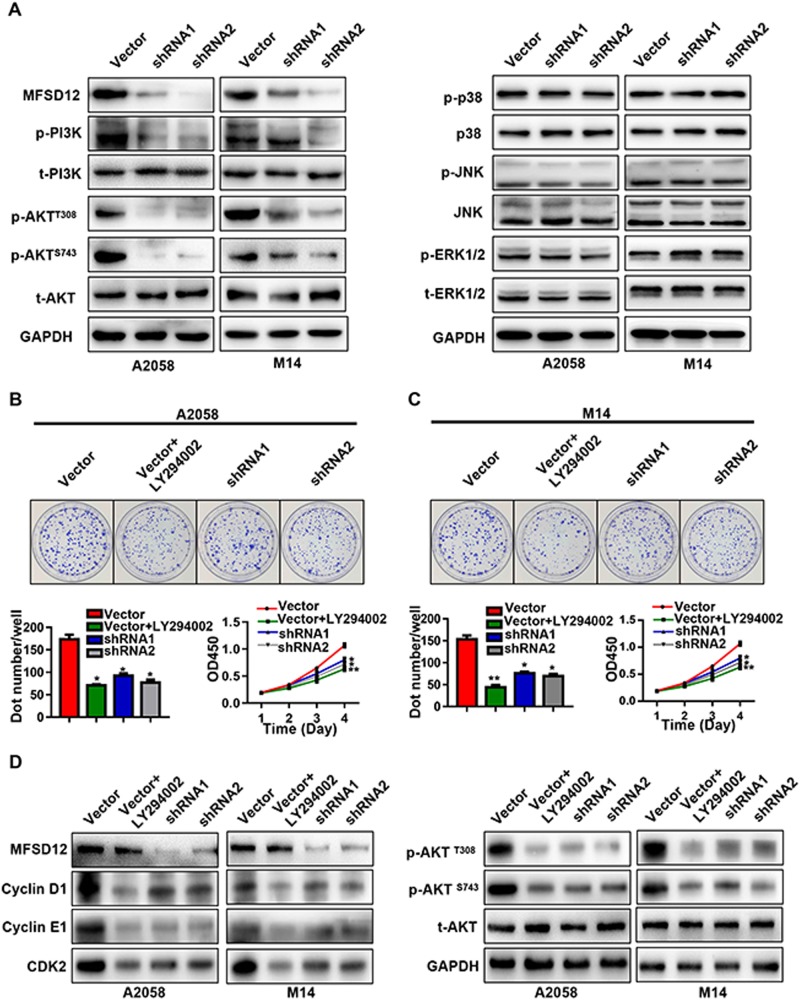
MFSD12 regulates melanoma cell proliferation via the PI3K–AKT signaling pathway. **a** Several signal molecules are presented in A2058-shRNA cells, M14-shRNA cells, and their control cells. **b** A2058-Vector cells treated with LY294002 compared with A2058-Vector cells not treated with LY294002; A2058-shRNAs applied to colony formation and CCK-8 assays. **c** M14-Vector cells treated with LY294002 compared with M14-Vector cells not treated with LY294002; M14-shRNAs applied to colony formation and CCK-8 assays. **d** Western blot bands for MFSD12, CDK2, cyclin D1, and cyclin E1; AKT signal molecules are shown in the indicated cells. GAPDH was used as the internal control. *p < 0.05; **p < 0.01

We further determined the molecular mechanism of MFSD12 in melanoma according to the GO and KEGG analyses (Fig. 5a). The results of these analyses showed that the levels of p-PI3K and p-AKT were decreased in MFSD12-shRNA cells compared with control cells and that the activation of MAPK signaling-related molecules did not change. To examine whether high expression of MFSD12 influences melanoma cell proliferation through the PI3K-AKT pathway, we treated A2058-Vector and M14-Vector cells with LY294002, a PI3K inhibitor (50 μM, 24 h). As presented in Fig. 5b, c, LY294002 prominently inhibited the proliferation of melanoma cells with a high level of MFSD12. Moreover, treatment with LY294002 downregulated p-AKT, CDK2, cyclin D1, and cyclin E1 in A2058-Vector and M14-Vector cells (Fig. 5d). These results suggest that a high level of MFSD12 promotes the proliferation of melanoma cells via the PI3K-AKT pathway.

### A high level of MFSD12 is associated with poor prognosis in melanoma patients

**Fig. 6:**
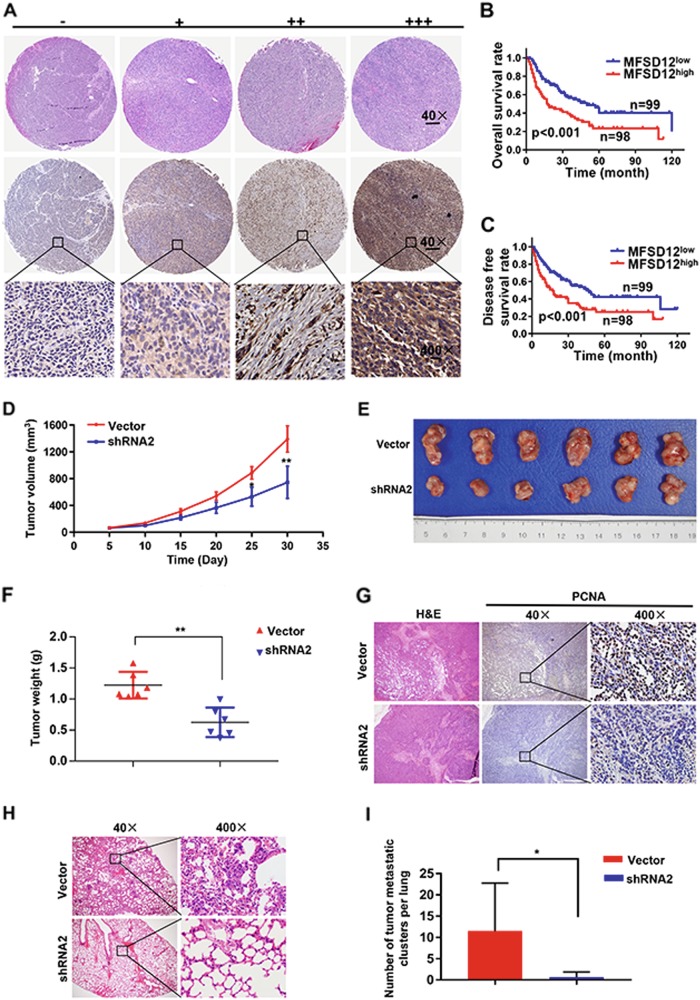
MFSD12 is associated with poor prognosis in melanoma patients and required for tumor growth and progression in vivo. **a** Representative images of tumor tissues in different staining classifications are shown and graded from “−” to “+++”. **b**, **c** Kaplan–Meier curves showing the relationship between MFSD12 expression and overall and disease-free survival. Scale bar, 40×, 500 μm; 400×, 50 μm. **d** Subcutaneous xenograft tumor volumes were measured at the indicated number of days; representative pictures are shown. **e**, **f** All the tumors are shown at the 30th day, and the weights are presented in the bar graph. **g** Subcutaneous xenograft tumors stained with H&E and IHC. **h**, **i** Representative H&E-stained metastatic clusters are shown; the number of tumor metastatic clusters per lung are presented in the bar graph. ^*^p < 0.05; ^**^p < 0.01

We demonstrated incredible heterogeneity of the MFSD12 protein in the tumor samples; a varying level of MFSD12 was detected in the tumor tissues (−, absent; +, weak; ++, moderate; +++, strong) and representative images were shown in Fig. 6a.

Then, the tumor samples were divided into two groups according to the expression of MFSD12, resulting in 98 patients with high expression and 99 patients with low expression. The relationship between the expression of MFSD12 and the clinical parameters of melanoma patients is displayed in Table 1. We found that high MFSD12 expression was positively correlated with advanced Clark level (*p* = 0.0039), distant metastasis (*p* = 0.0029) and advanced clinical stage (*p* = 0.027, Table 1).

**Table 1:** Correlations between MFSD12 with clinicopathologic features in 197 melanoma patients

Variable	Number of Patients		*P* value*
	MFSD12^low^	MFSD12^high^	
Age, year			
<60	41	37	0.600
≥60	58	61	
Gender			
Male	47	58	0.100
Female	52	40	
Anatomic site			
Acra	54	47	0.541
Trunk	24	24	
Other	21	27	
Histologic type			
Superfical spreading	22	24	0.136
Nodular	19	22	
Acral	42	27	
Lentigo maligna	16	25	
Ulceration			
Present	12	14	0.654
Absent	87	84	
Breslow depth (mm)			
≤2	58	55	0.727
>2	41	43	
Clark level			
I–III	61	46	0.039
IV–V	38	52	
Lymph nodes metastasis			
No	72	70	0.839
Yes	27	28	
Distant metastasis			
No	81	67	0.029
Yes	18	31	
Clinical stage			
I–II	70	54	0.027
III–IV	29	44	

Note: A chi-square test was used for comparing groups between low and high MFSD12 expression. *p < 0.05 was considered significant

The bold values was not necessary

By the end of follow-up, 106 (53.8%) patients had died. The overall 2- and 5-year survival rates were 57.5% and 32.1%, respectively. In addition, the MFSD12^high^ group had a lower rate of overall survival (OS) than the MFSD12^low^ group (*p* < 0.001, Fig. 6b). The 1- and 2-year OS were respectively 84.6% and 52.2% for the MFSD12^Low^ group, and only 37.1% and 15.9% for the MFSD12^High^ group. Moreover, the patients scored as MFSD12^high^ had significantly lower disease-free survival rates than the patients scored as MFSD12^low^ (*p* < 0.001, Fig. 6c). To better understand the prognostic value of MFSD12 in melanoma patients, we conducted a further analysis by dividing all the patients into several subgroups, and we observed that the predictive value of MFSD12 level for OS was maintained for the subgroups divided according to Breslow thickness (<5 mm, *p* = 0.002), Clark level (I–III, *p* = 0.039; IV–V, *p* = 0.019), absence of lymphatic metastasis (*p* < 0.001) and absence of distant metastasis (*p* = 0.009, Supplementary Figure S3A). For DFS, the predictive value of MFSD12 level was maintained for the subgroups divided according to Breslow thickness (<5 mm, *p* = 0.002), Clark level (I–III, *p* = 0.039; IV–V, *p* = 0.019), absence of lymphatic metastasis (*p* < 0.001) and absence of distant metastasis (*p* = 0.009, Supplementary Figure S3B).

The results of the univariate and multivariate analyses are shown in Table 2. The univariate analysis showed that Breslow thickness, Clark level, lymphatic metastasis, distant metastasis, and clinical stage, as well as MFSD12 staining, were associated with OS and DFS. In particular, the expression of MFSD12 had a great impact on OS and DFS time, and the average OS for the patients with low and high MFSD12 expression was 34.6 months and 22.4 months, respectively, and the average DFS was 31.6 months and 20.1 months, respectively. Subsequently, we verified the role of MFSD12 expression in prognosis by combining all the factors that were statistically significant in a univariate analysis to and performing a multivariate analysis. A high MFSD12 expression level represented a promising and independent prognostic variable for the prediction of melanoma progression. Other factors, including Clark level and clinical stage, were also assessed, and the results are presented. In conclusion, MFSD12 is a risk marker for OS and DFS in melanoma patients.

**Table 2:** Univariate and multivariate analyses of factors associated with OS and DFS

	OS multivariate Analysis				DFS multivariate analysis			
Variable	Univariate *p*	HR	95% CI	p*	Univariate *p*	HR	95% CI	p*
Age, year (≥60 vs. <60)	0.230			NA	0.286			NA
Gender (Men vs. Women)	0.741			NA	0.798			NA
Anatomic site (Acra vs. Trunk vs. Other)	0.858			NA	0.887			NA
Histologic type (Superfical spreading vs. Nodular vs. Acral vs. Lentigo maligna)	0.089			NA	0.054			NA
Ulceration (Present vs. Absent)	0.284			NA	0.249			NA
Breslow depth (mm) (≤2 vs. >2)	0.019			NS	0.017			NS
Clark level (I–III vs. IV–V)	0.014	1.646	1.107–2.448	0.014	0.027	1.588	1.067–2.362	0.023
Lymph nodes metastasis (Yes vs. No)	0.015			NS	0.015			NS
Distant metastasis (Yes vs. No)	0.002			NS	0.003			NS
Clinical stage (Yes vs. No)	<0.001	3.939	2.371–6.544	<0.001	<0.001	4.079	2.465–6.749	<0.001
MFSD12 staining (Low vs. High)	0.001	1.678	1.120–2.515	0.012	0.001	1.610	1.075–2.410	0.021

*OS* overall survival, *DFS* disease-free survival, *NS* not significant, *NA* not adopt

*, *p* < 0.05 was regarded as statistically significant, *p* value was calculated using Cox proportional hazards regression

### MFSD12 interference inhibits melanoma growth and progression in vivo

We further employed subcutaneous xenograft tumor models to identify the role and therapeutic implication of MFSD12 in melanoma. We found that downregulation of MFSD12 expression significantly inhibited the growth of xenograft tumors (Fig. 6d). The tumor sizes of A2058-Vector-derived xenografts were 1409 ± 54.48 mm^3^, significantly larger than those of xenografts originating from A2058-shRNA2 cells (712.5 ± 91.8 mm^3^) at the 24th day (Fig. 6e). Consistent with the tumor size, the tumor weights of A2058-Vector-derived xenografts were 1.225 ± 0.087 g, which were significantly larger than those of xenografts originating from A2058-shRNA2 cells (0.625 ± 0.097 g) at the 30th day (*p* = 0.0065, Fig. 6f). Moreover, PCNA was found to significantly downregulate in the A2058-shRNA2-derived xenografts compared with the A2058-Vector-derived xenografts (Fig. 6g). Importantly, the incidence of lung metastases was significantly increased in the nude mice implanted with A2058-Vector-derived cells compared with nude mice implanted with A2058-shRNA2-derived cells (Fig. 6h, i). These results indicate that MFSD12 upregulation promotes melanoma progression, and MFSD12 should be a promising target in the prevention and treatment of metastasis in melanoma.

## Discussion

Gene expression profiling and bioinformatics analysis have been widely used to identify potential diagnostic and therapeutic targets in different cancers [4, 5]. In the present study, the GSE3189 and GSE31879 profiles were selected for reanalysis, and a total of 140 overlapping DEGs, namely, 128 upregulated DEGs and 12 downregulated DEGs, were obtained. Some of these DEGs had been investigated previously, including CDK2, AKT1 and KIT [13, 14], which have been widely accepted as oncogenes in melanoma. Moreover, the PPI network analysis showed that AKT1 is located at the center of the network and is connected with multiple DEGs; other hub genes such as CDK2, KIT and CASP8 also connected with multiple genes, in agreement with the results of other studies [13, 15]. The GO and KEGG analyses showed that these DEGs are enriched in melanoma development-related pathways, for example, the KEGG pathways that are denoted pathways in cancers, PI3K–AKT signaling pathway, and Ras signaling pathway. Additionally, the expression levels of the 140 DEGs were verified and the log-rank test for overall survival and disease-free survival was conducted with GEPIA. Interestingly, only MFSD12 and SLC45A2 were found to be related to the patients’ OS and DFS. The reliability of the DEGs was also verified by qRT-PCR on melanoma tissues. These results showed the importance of the DEGs and confirmed the reliability of the gene expression profiling and bioinformatics analysis.

Oddly, both MFSD12 and SLC45A2 belong to the major facilitator superfamily, indicating that MFS may play important roles in melanoma. The MFS is the largest known superfamily of secondary carriers found in the biosphere [11]. This family is ubiquitously distributed throughout virtually all currently recognized organismal phyla and plays an important role in moving compounds across biological membranes [11, 16] during processes such as nutrient absorption and renal clearance [17]. Previous studies have demonstrated that the MFS transporters are responsible for multiple metabolic diseases [17, 18]. The most intensive studies are on the GLUT proteins, which play an essential role in maintaining whole-body glucose homeostasis; their dysfunction results in type 2 diabetes [2, 19]. Recently, the MFS transporters were reported to be associated with a variety of cancers. For example, Monica et al. [20] found that MFSD2A is a novel suppressor gene in lung cancer that acts on tumor growth and development through controlling the cell cycle profile, matrix attachment, and cell motility. Mitsuro et al. [21] identified MFSD4 as a putative tumor suppressor and biomarker for hepatic metastasis in gastric cancer patients. A recent paper reported that MFSD12 is associated with skin pigmentation, and functional analyses indicated that MFSD12 encodes a lysosomal protein that affected melanogenesis in zebrafish and mice [22]. Therefore, we speculated that MFSD12 may play a key role in melanoma and is of great significance.

Here, the MFSD12 mRNA expression in melanoma tissues was found to be significantly upregulated compared with that in normal tissues and in other cancers, suggesting that MFSD12 mRNA may be a novel diagnostic target with high sensitivity and specificity. In melanoma, the expression of MFSD12 was noticeably upregulated and the interference of MFSD12 expression in A2058 and M14 melanoma cells markedly decreased cell proliferation. Then, flow cytometric analysis was performed, and this analysis verified that silencing the expression of MFSD12 increased the percentage of cells in G1 phase but decreased the percentage of cells in S phase, which demonstrated that the MFSD12-induced proliferation was associated with promoting the G1-to-S phase transition. Western blot analysis and immunofluorescence staining further confirmed these results at the protein level. Correspondingly, we found that the MFSD12 mRNA expression level was positively correlated with the activation of CDK2 and cyclin D1, which have been widely accepted as biomarkers of the G1-to-S phase transition and play important roles in cell proliferation. Combining the KEGG pathway analysis and data from other reports, we further identified that the activation of the PI3K-AKT signaling pathway was responsible for the proliferation of melanoma cells, a result that was in line with those in other reports [23, 24]. Clinically, our survival curve revealed that melanoma patients with a high level of MFSD12 had a poorer prognosis than those with a low level of MFSD12 and that the expression of MFSD12 could be an independent prognostic factor for melanoma patients; these results were obtained with both univariate and multivariate analyses. Importantly, MFSD12 interference significantly weakened the tumor growth and metastasis in vivo, which further highlighted the important role of MFSD12 in melanoma progression. Moreover, the expression of MFSD12 was positively correlated with PCNA, a widely accepted biomarker for cell proliferation [25]. From the abovementioned results, it can be concluded that MFSD12 plays a key role in melanoma progression and may serve as a promising target for the prevention and treatment of metastasis in melanoma.

In conclusion, the expression of MFSD12 was markedly upregulated in melanoma and the elevated MFSD12 promoted melanoma progression by inducing cell proliferation via the PI3K–AKT pathway. High level of MFSD12 is a poor prognostic factor for melanoma patients and can serve as a potential therapeutical target of melanoma.

## Materials and methods

### Microarray data

We selected the GSE3189 and GSE31879 profiles from the GEO database. GSE3189, which was based on the Agilent GPL96 platform (HG-U133A, Affymetrix Human Genome U133A Array), included 45 melanoma and 18 nevus samples, and GSE31879, which was based on the Agilent GPL570 platform (HG-U133_Plus_2, Affymetrix Human Genome U133 Plus 2.0 Array), included 9 melanoma and 4 nevus samples. GEO2R (https://www.ncbi.nlm.nih.gov/geo/geo2r/) was applied to detect differentially expressed genes between the melanoma and nevus samples [26]. GEO2R is an interactive online tool that allows users to compare two or more groups of samples in a GEO series. The adjusted *p* values were utilized to reduce the false positive rate using the Benjamini and Hochberg false discovery rate method by default. An adjusted *p* value <0.01 and |logFC| ≥ 1 were set as the cutoff criteria. Venn (http://bioinformatics.psb.ugent.be/webtools/Venn/) is an online tool that can calculate the intersection(s) of lists of elements; this tool was used to obtain the overlapping DEGs in these two databases.

### PPI network, GO, and KEGG pathway analysis of DEGs

Search Tool for the Retrieval of Interacting Genes (STRING, https://string-db.org/) is an online tool designed to evaluate protein–protein interaction information [27]. To detect the potential relationships among the DEGs, we used the STRING app in Cytoscape and mapped the DEGs into STRING. The Database for Annotation, Visualization and Integrated Discovery (DAVID, https://david.ncifcrf.gov/) is a web-based bioinformatics resource that aims to provide tools for the functional interpretation of large lists of genes or proteins [28]. The Gene Ontology (GO) and Kyoto Encyclopedia of Genes and Genomes (KEGG) pathway analyses of the DEGs were performed with DAVID.

### Expression level of DEGs and survival analysis

GEPIA (http://gepia.cancer-pku.cn/index.html) was used to analyze the RNA sequencing expression data for 9736 tumor and 8587 normal samples from TCGA (The Cancer Genome Atlas) and the GTEx (Genotype-Tissue Expression) project in accordance with a standard processing pipeline [29]. GEPIA provides a customizable function for analyzing the association between DEGs and the overall survival (OS) and disease-free survival (DFS) of patients. Box plots and survival plots were constructed to visualize the expression levels of the DEGs and their association with prognosis. The p-values were calculated and are shown on the plots.

### Patients and follow-up

Thirty pairs of fresh frozen tumor and matched peritumor samples randomly collected from the Department of Plastic Surgery at Zhongshan Hospital of Fudan University (Shanghai, China) were analyzed by western blotting and qRT-PCR. A total of 138 paraffin-embedded melanoma and matched peritumor tissues and an additional 59 melanoma tissues were collected to construct the tissue microarray (TMA). All patients were subjected to a complete excision followed by tissue verifcation through pathological examination. The clinical and prognosis data were collected from 1 January 2008, to 31 December 2017. Prior to the surgery, no one had received any form of radiotherapy or chemotherapy, and detailed clinicopathological and follow-up data had been obtained from them. The clinical stage of the patients was evaluated by the TNM staging system of the American Joint Committee on Cancer (AJCC) and IUCC (7th edition) [30] at the time of the formal pathology report. Ethical approval for the study was obtained from the Ethics Committee of the Zhongshan Hospital Biomedical Research Department, and written informed consent was obtained from each patient.

### Cell culture and transfection

The melanoma cell lines A375, A2058, A875, SK-MEL-28, MV3 and M14 were purchased from the Cell Bank of the Chinese Academy of Sciences (Shanghai, China) and were grown under the recommended conditions. The vectors of pGMLV-SC5-Puromycin-EGFP-shRNA-MFSD12 and pGMLV-SC5-Puromycin-EGFP were transfected into A2058 and M14 cells and were purchased from Shanghai Genomeditech Company (Shanghai, China), according to the manufacturer’s instructions. The shRNA1 sequence is TGACCACCAGGCTCATCGT (forward) and ACGATGAGCCTGGTGGTCA (reverse) and the shRNA2 sequence is GTGATCTTCCAGTTTGGCT (forward) and AGCCAAACTGGAAGATCAC (reverse). Subsequently, the cells with suitable fluorescence expression were screened with puromycin at a concentration of 4 μg/ml. The transfection efficiency was verified by qRT-PCR and western blotting.

### Tissue microarray (TMA) construction and immunohistochemical staining

The tissue microarray was constructed as previously described [31]. The procedure of for the MFSD12 IHC was as performed in previous studies [32], and the primary antibody against MFSD12 is shown in Supplementary Table S1. Briefly, the slide was deparaffinized, rehydrated, antigen-retrievaled, and incubated in 0.3% H_2_O_2_ following the manufacturer’s instructions. Subsequently, the section was incubated with the primary antibody at 4 °C overnight, and then stained with Horseradish peroxidase-labeled IgG (Gene Tech, China). After that, the section was stained with diaminobenzidine, counterstained with hematoxylin. The TMA was viewed independently by two pathologists who were unaware of the patients’ clinical information, and disagreements were solved by reaching a consensus. The criteria for positive staining were assigned as previously described [33]. The density level of MFSD12 was defined by the intensity and percentage of positive staining in the whole cylinder as previously described [34]. The cells were scored by percent MFSD12-positive staining into five groups: 0 (0%), 1 (1 to ≤25%), 2 (25 to ≤50%), 3 (50 to ≤75%) and 4 (>75%). Groups 0, 1, and 2 were defined as having low expression, while groups 3 and 4 were defined as having high expression.

### Real-time reverse transcription PCR (qRT-PCR) and western blot analysis

Western blotting was performed as in a previous study [35], and all the primary antibodies are listed in Supplementary Table S1. Total RNA was extracted from cell lines and frozen tissues by TRIzol reagent (Invitrogen, USA), and reverse-transcribed to cDNA with a PrimeScript RT Reagent Kit (TaKaRa, Japan). The qRT-PCR primers are shown in Supplementary Table S2.

### BrdU, CCK-8, and colony formation assays

The BrdU incorporation assay is designed to quantitate cell proliferation based on the measurement of BrdU incorporation during DNA synthesis in proliferating cells. The developed color, and therefore the absorbance values, directly correlate with the number of proliferating cells in the respective microcultures. The BrdU assay was performed as in previous research [36]. Briefly, cells grown on coverslips were incubated with BrdU (Sigma, Germany) for 30 min and stained with anti-BrdU antibody (Abcam, UK) according to the manufacturer’s instructions. Images were acquired with a laser scanning microscope (Olympus, Japan).

The CCK-8 assay was performed as in a previous study [31]. Cells were inoculated into 96-well plates (1000 cells/well). At each time point (1st, 2nd, 3rd, and 4th day), 10 μl of CCK-8 solution was added to the sextuplicate wells. The plates were incubated for 3 h, and the absorbance of each well was determined at 490 nm.

The colony formation assay was performed as in a previous study [35]. Cells were seeded in a six-well plate (1000 cells/well) with the culture medium refreshed every 3 days for 2 weeks. Following the 2-week period, the cells were washed with PBS, fixed with 4% paraformaldehyde and stained with 0.4% crystal violet for 15 min. The number of colonies containing >10 cells was counted manually and averaged over the duplicate wells.

### Flow cytometric analysis and immunofluorescence staining

Flow cytometric analysis was performed as in a previous study [36] and was used to determine the cell cycle phase and to evaluate cell proliferation. The cells were washed with PBS and fixed in 70% ethanol overnight at 4 °C. Subsequently, the cells were stained with propidium iodide (BD Biosciences, USA) for 30 min and then detected by a flow cytometer.

Immunofluorescence staining was used to investigate the expression of MFSD12, CDK2, cyclin D1, and cyclin E1 in melanoma cells, as described previously [37]. Briefly, after treatment with 0.1% Triton X-100 for 30 min at 25 °C, the cells were blocked with 5% bovine serum albumin and incubated with the primary antibodies overnight at 4 °C. After three washes with PBS, the cells were incubated with secondary antibodies for 2 h. Finally, cell nuclei were stained with DAPI and imaged with a fluorescence microscope (Olympus, Japan).

### In vivo tumorigenesis and metastasis

Xenograft experiments in nude nice were approved by Animal Experimentation Ethics Committee of Zhongshan Hospital, Fudan University. Male with age from 4-6 weeks of BALB/c nude mice were maintained and handled in accordance with the stated guidelines of 3Rs (replacement, reduction, and refinement). All of them were randomized and blinded to the group assignment. These mice were randomly divided into two subgroups (*n* = 6/each group): a control group (Vector) and a treatment group (shRNA2). A total of 5 × 10^6^ cells (per mouse) were injected to establish subcutaneous xenograft tumor models as previously described [38]. Tumor growth was monitored every 5 days, and mice were sacrificed after 30 days. Tumor and lung tissues were fixed in formalin and embedded in paraffin. Consecutive sections were prepared for each lung tissue block and were stained with hematoxylin and eosin. The tumor volume was measured using the following formula: *V* = *π*/6 × (larger diameter) × (smaller diameter)^2^. The presence of lung metastases was calculated and evaluated independently by two pathologists.

### Statistical analysis

All experiments were performed in triplicate. The data were analyzed using IBM SPSS Statistics 20 (IBM Corp., USA). The values are expressed as the mean ± standard deviation. Student’s *t*-test was used for comparisons between groups. Categorical data were analyzed by the chi-square test or Fisher’s exact test. Correlation between two groups was determined by analysis of Pearson’s correlation coefficient. The DFS and OS rates were analyzed using the Kaplan–Meier method and the log-rank test. Cox’s proportional hazards regression model was used to analyze the independent prognostic factors. A *p*-value of <0.05 was considered to indicate a statistically significant difference.

## Electronic supplementary material

Supplementary Figure S3

Supplementary Figure S1

Supplementary Figure S2

Supplementary Figure legends

Supplementary Table S1

Supplementary Table S2
