# Supplementary figures and images for: Bioinformatics-based analysis reveals elevated MFSD12 as a key promoter of cell proliferation and a potential therapeutic target in melanoma
Source: Oncogene. 2018 Nov 1;38(11):1876–91. doi: 10.1038/s41388-018-0531-6 (PMC6462865; doi:10.1038/s41388-018-0531-6)

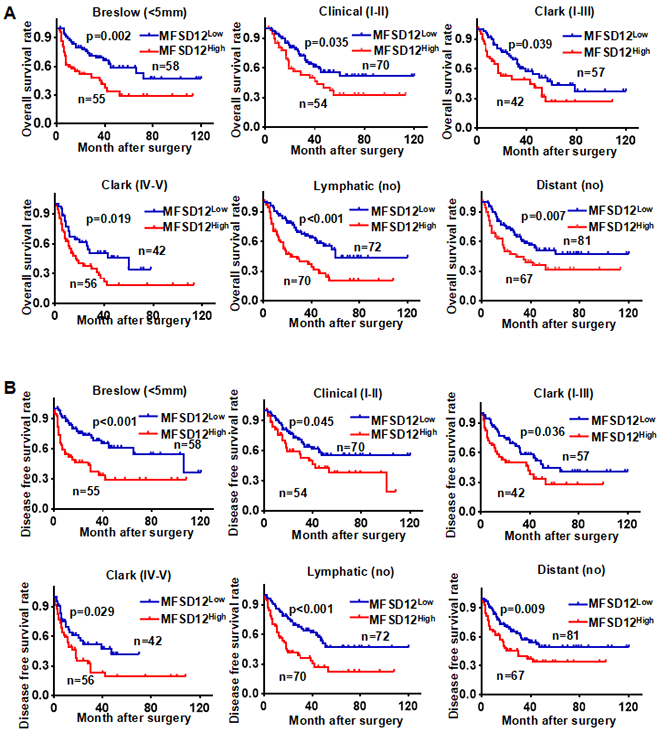

Supplement: Supplementary file 1 — Supplementary Figure S3 [file 41388_2018_531_MOESM1_ESM.tif]

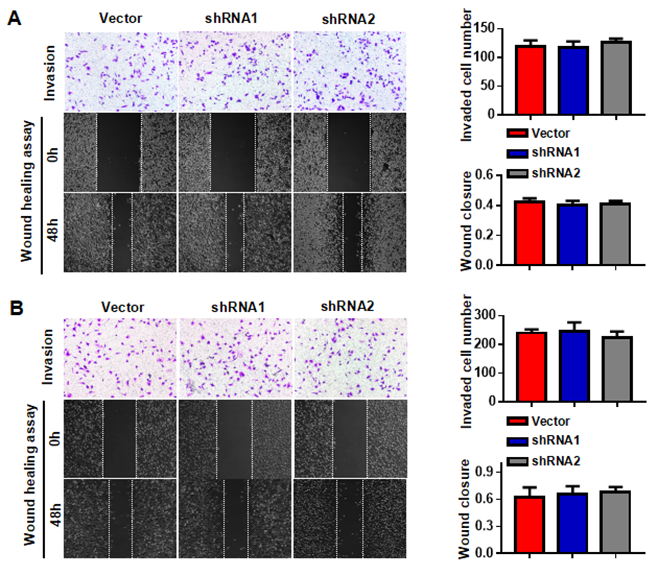

Supplement: Supplementary file 2 — Supplementary Figure S1 [file 41388_2018_531_MOESM2_ESM.tif]

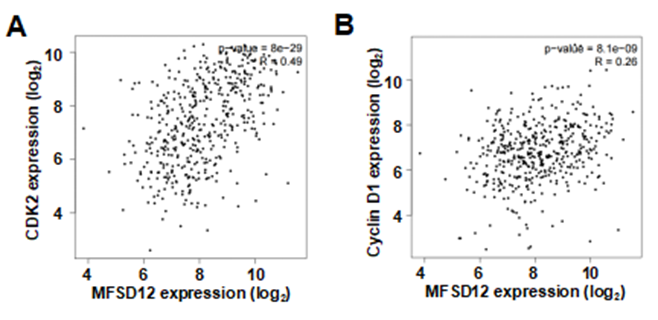

Supplement: Supplementary file 3 — Supplementary Figure S2 [file 41388_2018_531_MOESM3_ESM.tif]
