# Supplementary material for: Bioinformatics-based analysis reveals elevated MFSD12 as a key promoter of cell proliferation and a potential therapeutic target in melanoma
Source: Oncogene. 2018 Nov 1;38(11):1876–91. doi: 10.1038/s41388-018-0531-6 (PMC6462865; doi:10.1038/s41388-018-0531-6)
Supplement: Supplementary file 4 — Supplementary Figure legends [file 41388_2018_531_MOESM4_ESM.docx]

**Supplementary Figure S1.** **There is no effect on the invasion and migration of melanoma cells**

(**A**) Invasion and wound healing assays for shRNA cells and 2058-Vector cells. (**B**) Invasion and wound healing assays for shRNA cells and M14-Vector cells.

**Supplementary Figure S2.** **MFSD12 is associated with proliferative biomarkers**

1. Association of MFSD12 with CDK2 by GEPIA analysis. (**B**) Association of MFSD12 with cyclin D1 by GEPIA analysis.

**Supplementary Figure S3.** **MFSD12 is associated with poor prognosis in melanoma patients**

**(A)** Correlation of overall survival with MFSD12 expression in the Breslow thickness (<5 mm), clinical stage (I-II), Clark level (I-III), Clark level (IV-V), lymphatic metastasis (no), and distant metastasis (no) subgroups. **(B)** Correlation of disease-free survival with MFSD12 expression in the Breslow thickness (<5 mm), clinical stage (I-II), Clark level (I-III), Clark level (IV-V), lymphatic metastasis (no), and distant metastasis (no) subgroups.
