# Supplementary material for: Bioinformatics-based analysis reveals elevated MFSD12 as a key promoter of cell proliferation and a potential therapeutic target in melanoma
Source: Oncogene. 2018 Nov 1;38(11):1876–91. doi: 10.1038/s41388-018-0531-6 (PMC6462865; doi:10.1038/s41388-018-0531-6)
Supplement: Supplementary file 5 — Supplementary Table S1 [file 41388_2018_531_MOESM5_ESM.docx]

**Supplementary Table S1 List of Primary Antibodies Used In the study**

| Antibody | Applications | Company |
| --- | --- | --- |
| MFSD12 | WB, IHC-P, P-ELISA | Biorbyt (orb1989) |
| CDK2 | WB, IF, IHC | Abcam (ab32147) |
| CDK1 | WB, IF, IHC | Abcam (ab18) |
| Cyclin D1 | WB, IP, IF | Abcam (ab40754) |
| Cyclin E1 | WB, IP, IF | Abcam (ab74276) |
| Cyclin B1 | WB, IP, IF, IHC | Abcam (a2949) |
| GAPDH | WB, IP, IF, IHC | Abcam (ab8245) |
| p-Akt^T308^ | WB, IHC | Abcam (ab38449) |
| p-Akt^S473^ | WB, IHC, IF, ICC | Abcam (ab81283) |
| Akt | WB, IHC, ELISA, | Abcam (ab8805) |
| p-PI3K | WB, IP | CST (4228S) |
| PI3K | WB, IP | CST (4257S) |
| PCNA | WB, IHC, ICC | Huabio (R1306-5) |

**Abbreviations:** WB, western blot; IHC, immunohistochemistry; IF, immunofluorescence; IP, immunoprecipitation; ELISA, enzyme linked immunosorbent assay
