# Supplementary material for: Bioinformatics-based analysis reveals elevated MFSD12 as a key promoter of cell proliferation and a potential therapeutic target in melanoma
Source: Oncogene. 2018 Nov 1;38(11):1876–91. doi: 10.1038/s41388-018-0531-6 (PMC6462865; doi:10.1038/s41388-018-0531-6)
Supplement: Supplementary file 6 — Supplementary Table S2 [file 41388_2018_531_MOESM6_ESM.docx]

**Supplementary Table S2 Sequences of Primer for Real-time Polymerase Chain Reaction**

| **MFSD12** |  |
| --- | --- |
| Forward | 5’-ACAACGTGTGCAAGGACTGC-3’ |
| Reverse | 5’-GAGCTGGTTGAGGACGGTCT-3’ |
| **FAM174B** |  |
| Forward | 5’-CCTTGGTGACCCGCATTTC-3’ |
| Reverse | 5’-GTAAAGGCGAACGCCACGA-3’ |
| **MAD1L1** |  |
| Forward | 5’-GAGATGAGAGAGACCAACGGG-3’ |
| Reverse | 5’-GCTCAACCACGAATCTGGAAA-3’ |
| **SCARB1** |  |
| Forward | 5’-AATAAGCCCATGACCCTGAAGC-3’ |
| Reverse | 5’-GCCCCACATGATCTCACCC-3’ |
| **SEMA6A** |  |
| Forward | 5’-AACTAATGCCTTCAACCCTTCC-3’ |
| Reverse | 5’-TCCGGTAAATGACTGCGTCAA-3’ |
| **SLC45A2** |  |
| Forward | 5’-CTGGCCGCCACATCTATAAAT-3’ |
| Reverse | 5’-GTAGCAGAACTCTCTTCCGAAC-3’ |
| **TBC1D16** |  |
| Forward | 5’-TCCAAGAACAATGTCTGCGTG-3’ |
| Reverse | 5’-GCTCCCAGCATCTCATCCTT-3’ |
| **GAPDH** |  |
| Forward | 5’-GGTATGACAACGAATTTGGC-3’ |
| Reverse | 5’-GAGCACAGGGTACTTTATTG-3’ |
